# Tumorigenesis as a process of gradual loss of original cell identity and gain of properties of neural precursor/progenitor cells

**DOI:** 10.1186/s13578-017-0188-9

**Published:** 2017-11-07

**Authors:** Ying Cao

**Affiliations:** Model Animal Research Center and MOE Key Laboratory of Model Animals for Disease Study, Nanjing University, 12 Xuefu Road, Pukou High-Tech Zone, Nanjing, 210061 China

**Keywords:** Carcinogenesis, Cell fate, Cell lineage, Epithelial–mesenchymal transition (EMT), Neural development, Neural cells, Oncogene, Tumor suppressor gene, Tumorigenesis

## Abstract

Cancer is a complex disease without a unified explanation for its cause so far. Our recent work demonstrates that cancer cells share similar regulatory networks and characteristics with embryonic neural cells. Based on the study, I will address the relationship between tumor and neural cells in more details. I collected the evidence from various aspects of cancer development in many other studies, and integrated the information from studies on cancer cell properties, cell fate specification during embryonic development and evolution. Synthesis of the information strongly supports that cancer cells share much more similarities with neural progenitor/stem cells than with mesenchymal-type cells and that tumorigenesis represents a process of gradual loss of cell or lineage identity and gain of characteristics of neural cells. I also discuss cancer EMT, a concept having been under intense debate, and possibly the true meaning of EMT in cancer initiation and development. This synthesis provides fresh insights into a unified explanation for and a previously unrecognized nature of tumorigenesis, which might not be revealed by studies on individual molecular events. The review will also present some brief suggestions for cancer research based on the proposed model of tumorigenesis.

## Background

Tumorigenesis is the gain of malignant properties in normal cells, including primarily dedifferentiation, fast proliferation, metastasis, evasion of apoptosis and immunosurveillance, dysregulated metabolism and epigenetics, etc., which have been generalized as the hallmarks of cancer [1]. Molecular studies have identified enormous amount of somatic gene mutations, when considering around 30,000 somatic mutations in *TP53* alone [2], that could be related to these malignant properties in cancer cells. Mutations in oncogenes and tumor suppressor genes might cause these genes to change their expression levels or activities that could eventually lead to neoplastic transformation in normal cells. There are more than 3000 genes [3], including the classical oncogenes and tumor suppressor genes, that have been considered as ‘cancer related’ because of changes in their gene sequences or their expression levels/activities in cancer. Some theories, hypotheses and concepts have been put forward to establish a unified connection between these cancer related genes, gene mutations and the acquirement of cancer properties in cells. However, each of them cannot provide an exclusive explanation for tumorigenesis because of some inconsistencies [4, 5]. Epithelial–mesenchymal transition (EMT) is such a concept that seems to link gene expression changes during tumorigenesis and cancer malignant properties, but it has been challenged by some studies. Our recent research demonstrates that solid cancer cell lines exhibit properties of neural precursor/progenitors cells and the function/expression of cancer related genes in cancer are tightly correlated with their function/expression in embryonic tissues during embryogenesis, establishing the correlation between tumorigenesis and specification/development of a particular tissue type [6]. The correlation might provide a general mechanism for cancer development and suggests that EMT in cancer might be a misinterpretation. In the review, I will gather further evidence from literatures that provide additional supports for our proposal.

## EMT: a flawed concept in cancer

EMT is a fundamental process for gastrulation and tissue morphogenesis during normal development, and has been considered to play also an essential role during carcinogenesis. EMT is generalized as a phenotypic change, in which a polarized epithelial cell loses its polarity and adhesion with neighboring cells, and assumes a mesenchymal cell phenotype with a motile property. EMT process and the underlying mechanisms have been comprehensively investigated and reviewed extensively in literatures [7–17]. The earliest EMT event occurs during gastrulation during which the primary mesenchyme, or the mesoderm, is induced from the upper epiblast epithelium. Induction of parietal endodermal cells from primitive endodermal cells involves EMT. With the progress of embryonic development, EMT occurs for the formation of neural crest, which originates from the ectodermal cells locating between neural plate and epidermal ectoderm and is the precursor tissue for mainly the peripheral and enteric nervous systems and melanocytes. During further developmental process, EMT is involved in the formation of sclerotome mesenchyme, or the secondary mesenchyme, from the ventral somite, the formation of muscle from the more dorsal part of the somite, and the formation of endocardium, liver, pancreas, prostate, etc. [14, 16, 18]. Therefore, EMT occurs in tissues or organs that are derived from all three germ layers. Although epithelial and mesenchymal cells can originate from different lineages, they are usually distinguished by the expression of a few markers. While CDH1 is the most commonly used marker for epithelial cells, expression of SNAI1, SNAI2, TWIST1, VIMENTIN, ZEB1, ZEB2, etc., identifies mesenchymal cells and promotes a mesenchymal phenotype.

EMT has been employed to explain carcinogenesis due to a few simple analogies between EMT and cancer progression. Most solid cancer types are of epithelial origin. During both developmental EMT and carcinogenesis, cells lose their polarity and adhesive properties, and acquire motility. The phenotypic change of cells undergoing EMT is accompanied by the loss or downregulation of epithelial specific genes and gain or upregulation of mesenchymal genes. This trend of marker expression change also occurs during cancer development. Accompanied with the trend of marker expression change is the acquisition of malignant features in cancer cells, including unlimited cell proliferation, evasion of cell death and immunosuppression, chemoresistance, genomic instability, stemness, etc. [13, 19–22]. Thus, ‘EMT’ has long been considered as the stimulus for epithelial cells to acquire the properties of malignancy. Nevertheless, there are serious flaws in the link between ‘EMT’ and carcinogenesis. Carcinomas originate from epithelial cells of various tissues or organs that are derived from all three germ layers and their lineages, for example, liver from endoderm, kidney from mesoderm and skin from ectoderm. This means that, besides expression of general epithelial markers, typically CDH1, epithelial cells of different tissues or organs should also express genes that are specific to tissue types or organs, including tissue- or organ-specific differentiation factors or genes. Despite that CDH1 is expressed in the epithelial cells of liver, kidney or skin, there are definitely specific genes that distinguish the epithelial cells in these organs. Similarly, the term ‘mesenchymal cell’ also represents a collective description of cells with mesenchymal properties derived from different lineages, which express not only the mesenchymal markers, but also lineage-specific factors. In fact, the process of cancer initiation and progression includes gradual expression change of many genes, in addition to the EMT marker genes. Amusingly, expression change of these tissue-/organ-specific genes and other genes has been not considered in cancer ‘EMT’. One possible reason might be that gene expression change during cancer initiation and progression was not well understood when the EMT concept was introduced to cancer research. Tumorigenesis has been considered as a process of dedifferentiation and reprogramming of somatic cells [23–27], which means the loss of differentiation markers and gain of progenitor/stem cell markers [25]. If these changes had been taken into account, then carcinogenesis should have not been merely considered as a result of loss of epithelial property. Although EMT is represented by similar expression change of a same set of markers and by the same cellular phenotypic alteration, it is said that developmental EMT events in blastula formation, gastrulation, neural crest formation, somitogenesis and endocardium formation are not associated with cancer, whereas those in trophoblast invasion, mesothelium, liver and prostate formation are considered as cancer-associated [16]. This means that EMT in different cellular context has different effects on cell physiological functions, and this adds more confusion to roles of EMT during carcinogenesis. Importantly, how EMT contributes to cancer cell malignant features has been not understood so far, except the knowledge about a handful of EMT markers and their universal expression change during cancer development. Since it has been widely accepted as a principle that EMT plays a central role during carcinogenesis, mechanistic studies on cancer EMT are almost exclusively concentrated on the regulation of EMT markers, for example, by transcription factors and signaling pathways, epigenetic factors, posttranslational modification factors, non-coding RNAs, etc. [13, 17, 28–37]. To compromise the situation, the content of cancer ‘EMT’ concept has thus been refined and some features like ‘EMT plasticity’, ‘partial EMT’, ‘intermediate EMT’, or ‘hybrid epithelial/mesenchymal’, etc., are introduced due to that transition from epithelial to mesenchymal state is a multi-step, multi-state, and dynamic process, ranging from an entirely epithelial to an entirely mesenchymal phenotype [34, 38–43]. A simplified explanation for this refinement could be that a 50% reduction of CDH1 expression with a mild CDH2 expression means a partial EMT, whereas a 100% reduction with a strong CDH2 expression represents a complete one. The intermediate states of EMT have also been reported for embryonic development and cancer development [42, 44–46]. However, the key point of these new features still relies on the expression of classical EMT markers and on the mechanisms for establishing intermediate levels of EMT marker expression [34, 38, 40, 42]. Therefore, how the expression levels of EMT markers are associated with malignancy, such as cancer cell stemness, is still mysterious [38, 41]. In fact, there is not a type of mesenchymal cells, except neural crest cells, displaying malignant properties other than cell mobility. Besides, a few other reports also cast doubts about the role of EMT during carcinogenesis. Tarin et al. [47] analyzed the incoherence within the relation between developmental EMT and cancer development, and emphasized that expression change of a few EMT markers is an oversimplification of neoplastic transformation from a healthy to a tumor cell. In mouse mammary tumor models, EMT effect was observed in tumor. However, it is not a prerequisite for invasiveness and metastasis in breast cancer [48]. By using mouse models of breast cancer and pancreatic cancer, separately, two more recent studies also have shown that EMT is not a relevant factor to drive metastasis, but rather a factor conferring chemoresistance to cancer cells [21, 22], although the two studies are also challenged [49, 50]. Studies of this kind have been rare; however, they raise serious concerns about the roles of EMT in cancer.

## A general correlation between gene expression/function in cancer and in specific embryonic tissues

If the involvement of EMT in cancer development and progression has been a misinterpretation, then what should be the real nature of cancer development? In an attempt to find a way to drive terminal differentiation of cancer cells, our recent work found a link between tumorigenesis and neural specification/development, shedding light on a unified understanding towards the nature of cancer initiation and development [6]. Based on some typical features of cancer cells, we were able to induce terminal differentiation of cell lines of different cancer types. Cancer cells are different from healthy cells in their behaviors and physiological functions, which are determined by the difference in global gene transcription. Cancer is therefore ultimately a disease of aberrant gene expression [51]. Cancer cells are immature cells resulting from dedifferentiation or reprogramming of somatic cells [23–27]. During neoplastic reprogramming, genes that promote cancer, such as those promoting cell cycle, stemness, survival, chemoresistance, migration, are activated or upregulated; whereas tumor suppressor genes and differentiation related genes including tissue- or organ-specific genes are silenced or downregulated in cancer cells. This transcriptional reprogramming involves a concerted regulation by transcriptional regulators, primarily transcription factors, transcriptional co-factors and epigenetic modification factors. The latter has drawn intensive attentions in recent years due to their central roles in neoplastic reprogramming of cells and cancer progression. These epigenetic modification factors include mainly the enzymes for DNA methylation/demethylation, histone acetylation/deacetylation, lysine methylation/demethylation, and arginine methylation/demethylation. The aberrant expression and functions of epigenetic modification factors during cancer development and progression have been extensively reviewed [52–59]. These enzymes also play extensive roles in the regulation of functions of non-histone proteins [60, 61]. Cancer is a disease of high heterogeneity, either between different cancer types or within a type of cancer. Nevertheless, if considering that different cancer types share a set of hallmarks [1], this implies that there might exist a shared mechanism to regulate these hallmarks that are common to cancer.

We tried to find out whether it was possible to drive terminal differentiation of cells from different cancer types using a same approach. Since we didn’t have technical screening strategies for identifying some common molecules that are responsible for differentiation/dedifferentiation of cancer cells of different cancer types, we tried to figure out by applying a few rules, which were inferred from the features of cancer cells, to the known epigenetic modification factors. The first rule is that the candidate factors should promote or be up-regulated in cancer. This emphasizes the common functions of the candidates among different cancer types. Considering that differentiation or tumor suppressor genes are usually silenced/downregulated in cancer, we were interested in the enzymes that mediate transcriptional repression, hoping that inhibition of the candidates could stimulate re-activation of these genes. The clues of the function of a gene in regulating differentiation of an immature cell could be found from the development defect phenotypes of early knockout embryos (gastrula or earlier) and/or its role in regulating embryonic stem (ES) cell differentiation. Phenotypes in later tissues or organs were not considered because this information might only reflect tissue-specific function of a gene. The number of candidate factors was still narrowed down by that they should be conserved in the basal species of multicellular organisms, such as *Amphimedon queenslandica*, because a major part of cancer related genes are conserved in unicellular and basal species of multicellular organisms [3] and conservation reflects their role in regulating basal differentiation events throughout evolution. These restrictions led us to focusing on HDAC1, HDAC3, EZH2, LSD1 and DNMT1, the best-known epigenetic modification enzymes. HDAC1/3 are class I histone deacetylases; EZH2 (also known as KMT6 or ENX-1) is the catalytic subunit of the PRC2 complex mediating trimethylation of histone H3 at lysine 27 (H3K27me3); LSD1 (also known as KDM1A, BHC110 or AOF2) regulates demethylation of histone H3 mono- or dimethylated at lysine 4 (H3K4me1/2); and DNMT1 is responsible for methylation of DNA CpG islands. It was not known whether there was a DNMT1 homologue in lower organisms when we performed the study. The latest data update for *Amphimedon queenslandica* shows that there is indeed a homologue in this species.

An intriguing result we achieved was that simultaneous inhibition of these enzymes led to a post-mitotic neuron like differentiation in most of the cancer cell lines we tested, including hepatocellular carcinoma, prostate cancer, breast cancer, colon cancer, melanoma, osteosarcoma, glioblastoma, and lung cancer. As expected, cancer cell lines after differentiation displayed a dramatically decreased expression in many tumor-promoting factors and lost malignant properties including proliferation, anchorage-independent growth, migration/invasion [6] in vitro assays. Chemical inhibitors of these enzymes also demonstrated strong a repressive effect on tumor formation in tumor cell xenograft assays and in an intestine tumor model [6].

This raises the question why blocking of a same set of epigenetic modification enzymes in different cancer cell lines can cause similar neuron-like differentiation. Because each of these enzymes regulates different chromatin modifications and non-histone proteins, it is very complex to analyze the exact molecular mechanisms underlying the differentiation effect. However, there are reports supporting their roles in regulating neurogenesis or neural development [62–64]. Regardless of the precise molecular mechanisms, the important information is that, similar to differentiation of a particular type of tissue or organ from their respective precursor/progenitor cells during embryonic development, neuron-like differentiation of distinct cancer cell lines provides the convincing evidence that cancer cells possess the potential for neuronal differentiation, a key property of neural precursor/progenitor cells. In agreement, the genes for all these enzymes demonstrate specific transcription in neural precursor tissues, i.e., the neural plate and neural crest during *Xenopus* neurulation, when tissue precursors are forming, and in primarily the nervous system later. This was possibly a hint that pan-cancer promoting genes might function in regulating the differentiation of a particular tissue during embryogenesis. The hint became more evident when the EMT mesenchymal marker genes were only detected in neural precursor cells, whereas the epithelial marker was in epidermal cells. The mutual exclusive expression of epithelial and mesenchymal marker genes makes EMT looked like a cell fate change from epidermal to neural cells during embryonic neural development. Specific transcription of these chromatin modification enzymes and mesenchymal makers in embryonic neural precursor/progenitor cells implied an intriguing correlation between the function/expression of a gene in cancer and the expression/function in a specific embryonic tissue type; however, this small number of genes was not enough to generalize such a relevance. Confirmation of this correlation needs to clarify how ‘cancer related genes’ are related with cancer. When more than 3000 cancer related genes were categorized according to their function/expression in different cancer types and in embryonic neural cells, the correlation became clear: an overwhelming majority of the genes promoting or being upregulated/activated in multiple cancer types (simply considered as tumor promoting genes, TPGs) shows specific expression in embryonic neural cells and neural crest cells, whereas genes repressing or being downregulated/silenced in multiple cancer types (simply considered as tumor suppressing genes, TSGs) tend to express more likely in non-neural cells. The genes that play dual roles, i.e. acting as a TPG in some cancer types while as a TSG in others, show almost equal chance with neural or non-neural expression. The correlation was also confirmed by our detection of expression patterns of some cancer related genes whose embryonic expression had not been reported. It is not surprising that the cancer genes with neural specific expression play extensive roles in all aspects of neural development, including differentiation, migration, maturation, neuritogenesis, axonal guidance, etc. This means that cancer-promoting genes should be able to prevent neurodegeneration. Actually, there has been epidemiologic and biological evidence showing an inverse association between cancer and neurodegenerative diseases [65]. Moreover, neurodegeneration caused by cancer therapy has been reported, as discussed below.

## Tumorigenesis resembles an uncontrolled process of neural specification/development

What does this correlation mean? EMT has been used to describe a phenotypic change from epithelial to mesenchymal type cells during cancer development. Our gene expression analysis demonstrated that EMT epithelial markers are among the TSGs with non-neural expression, and the mesenchymal markers are among the TPGs with neural specific expression. Therefore, cancer ‘EMT’ gene expression represents a minute part of the correlation between gene function/expression in cancer and in specific embryonic tissues. Hundreds of, but not just a few, cancer-promoting genes show specific expression in embryonic neural cells, neural crest cells and their lineages. Such a consistent cell type-specific expression was not discovered in other particular types of cells, especially mesenchymal cells. This demonstrates that cancer cells share regulatory networks with embryonic neural cells and suggests that these regulatory networks endow cancer cells with properties of embryonic neural cells rather than mesenchymal cells.

These shared genes between cancer cells and embryonic neural cells are involved in regulating all malignant properties, including increased cell cycle and proliferation (e.g., *AURKA*, *CCND1*, *CCND2*, *CCNB1*, *CCNE1*, *CDC25A*-*C*, *CDK1*, *CDK2*, *CDK4*, *CDT1*, *E2F1*, *PCNA*, etc.), angiogenesis (e.g., *FGFR1*-*4*, etc.), stemness (e.g., *MYC*, *POU5F1*, *SOX2*, *NESTIN*, *MSI1*, etc.), evasion of programmed cell death (e.g., *BIRC5*, *HSPA9*, *MNT*, etc.), dysregulated cell metabolism (e.g., *AKT*, *FUT4*, *KRAS*, *MTOR*, *MYC*, *OGT*, *PDK4*, *PFKP*, *PTK2*, etc.), chemoresistance (e.g., *IGF2*, *IGF2BP3*, *YAP1*, etc.), dysregulated epigenetics (*DNMT1*, *HDAC1*, *HDAC3*, *EZH2*, *LSD1*, *SETD1A*, *SETDB1*, *G9A*, *PRMT1*, *UTX*, *PRMT5*, *JMJD3*, *JMJD6*, *JHDM1A/B*, *WHSC1*, etc.). Although *GLUT1*, coding for a glucose transporter, and *ABCB1* and *ABCG2*, coding for two regulators of multidrug resistance, are not detected significantly in embryonic neural tissues, they serve as markers for neural stem cells [66–68]. Emphasized here is that neural crest cells and neural precursor/progenitor/stem cells, but not mesenchymal cells, exhibits the property of stemness. Besides, the major signal transducers in the TGFbeta, WNT/CTNNB1, FGF, NOTCH, HH, HIPPO/TAZ, IGF, HGF, PDGF, all being the EMT signaling pathway [13] and regulating all aspects of malignancy including immunosuppression, show specific expression in embryonic neural cells. STAT3 signaling pathway, a major regulator of cancer cell immunosuppression, regulates neuron differentiation and *stat3* show embryonic neural transcription. The functions of these signaling pathways during neural induction, i.e. induction of neuroectoderm from ectoderm, and subsequent neural development, have been well known. Noteworthy is that TGFbeta signaling must be inhibited during neural induction, as shown by neural differentiation of *Xenopus* ectoderm and embryonic stem cells in the absence of TGFbeta signaling [69–73], because it drives non-neural differentiation during germ layer formation [74, 75]. Similarly, inhibition of TGFbeta signaling is also a precondition for cancer initiation [76]. Upon neural induction and cancer initiation, it is involved in subsequent neural development and cancer progression [76], reinforcing the similarity in regulatory mechanisms between neural development and tumorigenesis. In addition to the neural genes and signaling pathways for which the expression/function in cancer are known, our work showed that cancer cells, e.g. HepG2, may express a much broader range of neural specific genes [6]. The expression/function of the additional neural genes are largely unknown in cancer yet, they are the components of the regulatory networks in neural cells. These analysis supports strongly that cancer cells are much more like neural cells than any mesenchymal-type cells. One may argue that each of the genes and signaling pathways discussed above could participate in regulating multiple events in multiple tissue or cell types, and different tissue or cell types may also exhibit one or two properties of malignancy, for example, cell mobility; however, when different cancer related properties and expression/function of the cancer-promoting genes and signaling pathways are integrated together, they point to one cell type, the neural cells.

Besides the shared regulatory networks, cancer cells are comparable to neural cells in their cellular morphology and behavior. Neural plate is the precursor tissue for the central nervous system, and neural crest gives rise to the peripheral nervous system. During neural development, neural plate-derived neuronal precursor cells and neural crest cells migrate from their places of origin into the places for their function, undergo extensive morphological changes, extend neurites towards their target cells under guidance, and innervate most tissues [77, 78]. Cancer cell morphological change, migration/invasion and metastasis to other tissues mimic well the neural development process. A comparison of cancer cell properties between cancer cells, epithelial-type cells, mesenchymal-type cells and neural cells is summarized in Table 1.

**Table 1:** Comparison of cancer cell properties between cancer, mesenchymal type, epithelial type and neural cells

Cancer cell properties, and genes or pathways regulating the properties	Cancer cells	Mesenchymal type cells	Epithelial type cells	Neural precursor/progenitor/stem cells
Marker genes^a^	Around 400 genes with upregulation/activation in cancer, including mesenchymal marker genes and neural progenitor/stem cell marker genes [6]	*CTNNB1*, *CDH2*, *SNAI1*, *SNAI2*, *TWIST1*, *VIMENTIN*, *ZEB1*, *ZEB2*	*CDH1*	*ABCG2*, *ASCL1*, *CTNNB1*, *BMI1*, *CDH2*, *CXCR4*, *FGFR2*, *FGFR4*, *FUT4*, *GLUT1*, *HES1*, *ID2*, *JAG1*, *JUN*, *MSI1*, *NESTIN*, *NOTCH1*, *PDGFRA*, *ROR2*, *SNAI1*, *SOX2*, *SOX3*, *SOX9*, *STAT3*, *VIMENTIN*, *ZIC3*, etc.
Mobility	Mobile, invasive, metastasizes to many tissues	Mobile	Immobile, adhesive	Migratory. innervate most tissues
Cell polarity	Loss of polarity upon tumor initiation	Loss of polarity	Polarized	Extensive morphological changes during neural development
Cell cycle/proliferation	*AURKA*, *CCND1*, *CCND2*, *CCNB1*, *CCNE1*, *CDC25A*-*C*, *CDK1*, *CDK2*, *CDK4*, *CDT1*, *E2F1*, *PCNA*, etc	No	No	*AURKA*, *CCND1*, *CCND2*, *CCNB1*, *CCNE1*, *CDC25A*-*C*, *CDK1*, *CDK2*, *CDK4*, *CDT1*, *E2F1*, *PCNA*, etc
Angiogenesis	*FGFR1*-*4*, etc	No	No	*FGFR1*-*4*, etc
Chemo-resistance	*ABCB1*, *ABCG2*, *IGF2*, *IGF2BP3*, *YAP1*, etc	No	No	*ABCB1*, *ABCG2*, *IGF2*, *IGF2BP3*, *YAP1*, etc
Stemness	*MYC*, *POU5F1*, *SOX2*, *NESTIN*, *MSI1*, etc	No	No	*MYC*, *POU5F1*, *SOX2*, *NESTIN*, *MSI1*, etc
Dysregulated epigenetics	*DNMT1*, *HDAC1*, *HDAC3*, *EZH2*, *LSD1*, *SETD1A*, *SETDB1*, *G9A*, *PRMT1*, *UTX*, *PRMT5*, *JMJD3*, *JMJD6*, *JHDM1A/B*, *WHSC1*, etc	No	No	*DNMT1*, *HDAC1*, *HDAC3*, *EZH2*, *LSD1*, *SETD1A*, *SETDB1*, *G9A*, *PRMT1*, *UTX*, *PRMT5*, *JMJD3*, *JMJD6*, *JHDM1A/B*, *WHSC1*, etc
Dysregulated metabolism	*AKT*, *FUT4*, *GLUT1*, *KRAS*, *MTOR*, *MYC*, *OGT*, *PDK4*, *PFKP*, *PTK2*, etc	No	No	*AKT*, *FUT4*, *GLUT1*, *KRAS*, *MTOR*, *MYC*, *OGT*, *PDK4*, *PFKP*, *PTK2*, etc
Apoptosis evasion	*BIRC5*, *HSPA9*, *MNT*, etc.	No	No	*BIRC5*, *HSPA9*, *MNT*, etc
Immuno-escape	STAT3 signaling, TGFβ signaling, WNT/CTNNB1 signaling, HIPPO/TAZ signaling, etc	No	No	STAT3 signaling, TGFβ signaling, WNT/CTNNB1 signaling, HIPPO/TAZ signaling, etc

^a^Here the marker genes for cancer cells do not refer to cancer biomarkers, but refer to the genes that are upregulated/activated or play a promoting role in cancer

But the analogy between cancer cells and neural cells does not mean that they are the same. The difference resides in that normal neural development is coordinately regulated by intrinsic/extrinsic signals in a balanced fashion in a neural specific environment, leading to neural formation in a correct spatiotemporal pattern, whereas tumorigenesis is a chaotic process. As discussed above, neural cells express high level of genes that promote proliferation, survival, high rate of metabolism, migration, etc. This means that neural cells are under high risks of gene mutations, unrestricted proliferation and survival, or misled cell migration, etc., which would eventually cause malformation of the nervous system, or even tumor formation, if not well controlled. This does not occur during normal neural development since the process is deliberately balanced by antagonistic signals, including the TSGs. Among TSGs with neural specific expression [6] during embryonic neurulation, *casp3*, *casp9*, *egln3*, *foxo4*, *gadd45* *g*, *nf2*, *pten* and *tp53* promote cell apoptosis or senescence; *apc*, *arid1a*, *cdkn1b*, *gadd45* *g*, *gas1*, *ndrg2*, *nf2*, *pbrm1*, *pdcd4*, *rb1*, *tes* and *tp53* inhibit cell cycle or proliferation; *pten* plays also a role in restricting migration and growth; *tp53* and the energy sensor gene *ampk* are responsible for balancing metabolic process; and *arid1a*, *pten* and *tp53* serve to maintain genomic integrity. Besides these internal antagonistic signals, external signals from neighboring non-neural cells antagonize neural development, for example, by inhibition of cell fate commitment, thereby restricting neural development within a correct region of an embryo. More generally, antagonism between signals from neighboring tissues is a mechanism that promotes cell fate specification or commitment, guarantees tissue or cell identity, establishes boundaries between different tissues, and ensures the formation of tissues in correct spatiotemporal patterns. These types of non-neural signals should be inactivated to promote neural development. Accordingly, we identified that a lot more TSGs are expressed in non-neural cells [6]. As they should be silenced in neural cells, they are silenced during tumorigenesis. There are typical examples for the tumor suppressor function of non-neural tissue-specific genes or genes for non-neural lineage specification factors. In addition to the epidermal protein CDH1, the endodermal tissue specification factors SOX17 and HHEX, and the myogenic factor MYOD, exhibit a suppressive effect on various types of cancer. There is evidence that RAS and MYOD repress each other’s function [79], reflecting exactly the antagonism between the signals from different tissue types because *kras* is specifically expressed in embryonic neural precursor/progenitor cells and *myod* is in muscle precursor/progenitor cells. Moreover, the cytoplasts without nucleus from non-tumorigenic rat myoblasts repressed the tumorigenicity of intact B16 mouse melanoma cells when they were fused to form cybrids [80]. It was interpreted that the suppressive effect of myoblasts was achieved by mitochondria in the cytoplasm [81]. However, the true principle behind might be the suppressive effect of non-neural factors on neural cells. The mitochondrial function is probably over-emphasized if considering likewise that reprogramming of a somatic cell nucleus into a pluripotent state by an enucleated oocyte is not an effect of mitochondrial function in the oocyte. Some closely related genes, such as those of a gene family, can serve as examples for the correlation between the function/expression in cancer and their tissue-specific expression in embryos. One example is the renowned *RAS* oncogene family members, *KRAS* and *HRAS*. Their products exhibit similar transformation activity; however, KRAS seems always to promote cancer, HRAS plays also a suppressive role or its expression is downregulated in cancer [82, 83]. Accordingly, *kras* is specifically expressed in embryonic neural cells, demonstrating its involvement in neural development. In contrast, *hras* is absent in these cells. Another example can be NOTCH1 and NOTCH2. In a mouse model of lung cancer, Notch1 promotes tumor initiation and progression, whereas Notch2 has tumor suppressor functions [84]. Their opposing functions in carcinogenesis correspond with neural specific expression of *notch1* [85] and non-neural expression of *notch2* [86] during embryonic neurulation. Additional examples include CDH2 versus CDH1, SOX2 versus SOX17, etc.

**Fig. 1:**
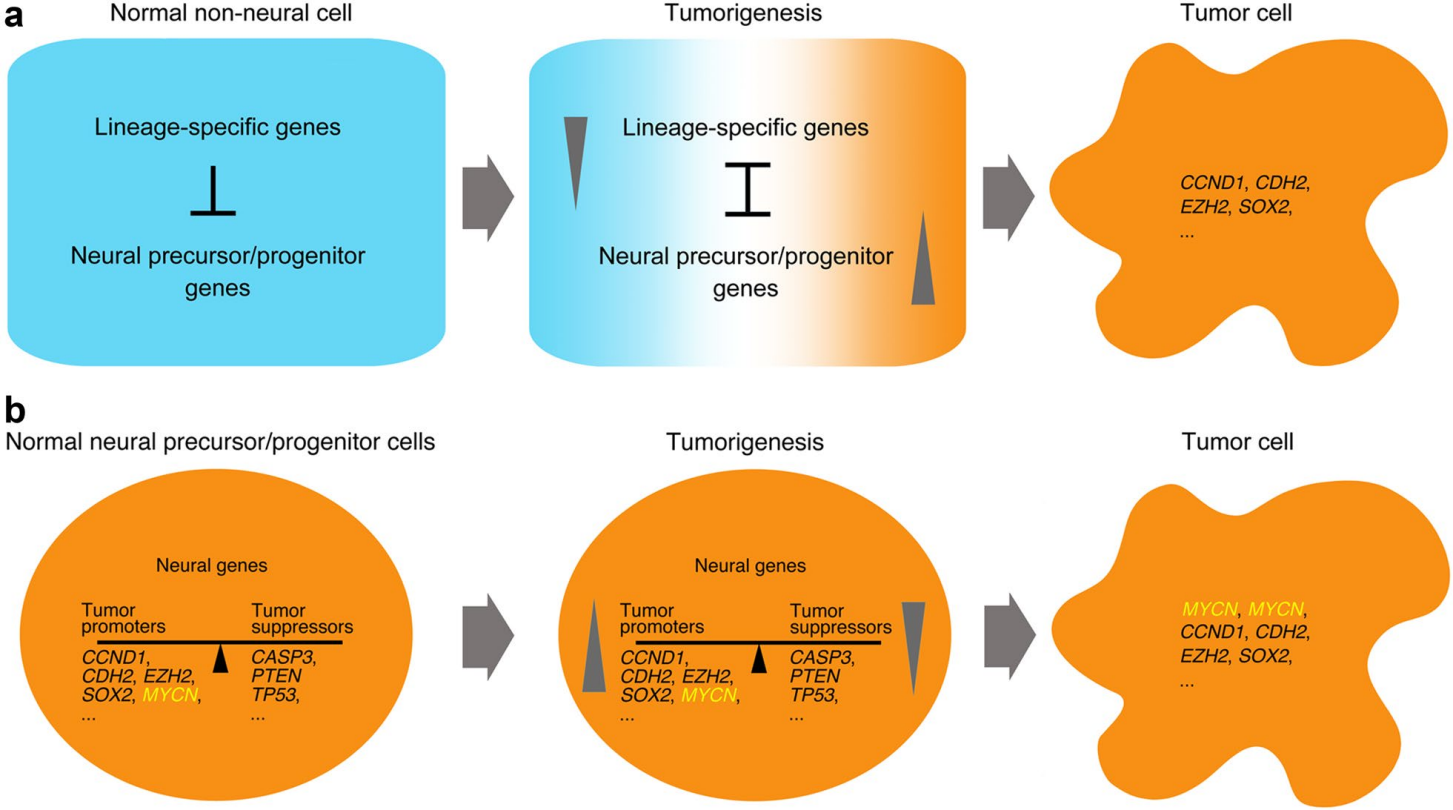
Models depicting the cancer development in non-neural and neural progenitor cells. **a** Tumorigenesis in non-neural cells. Normal non-neural somatic cells express lineage- or tissue-specific genes but without significant expression of neural specific genes. Some internal/external cellular changes may occasionally lead to activation/upregulation/gain-of-function of certain neural specific factors, which could cause the activation of subsequent signaling pathways required for neural specification/development. This activation of neural factors is accompanied by the suppression of lineage- or tissue-specific genes, hence, the loss of cell or lineage identity. Since the internal/external environment or regulatory mechanisms under this situation is rather imperfect for a normal neural specification/development, the cells undergo uncontrolled proliferation, migration, and even incomplete neuronal differentiation, which do not occur during normal neural specification/development. **b** Tumorigenesis in the nervous system. Tumors in the nervous system originate from neural progenitor cells [88], which harbor both promoting and inhibitory signals for proliferation, migration, differentiation, etc., so as to keep normal neural development in a balanced fashion. When the balance is broken, for example, by amplification of *MYCN*, tumorigenesis may occur

The correlation between the function/expression of cancer related genes and their embryonic tissue-specific expression might reveal a hidden nature of tumorigenesis, i.e., a process of gradual loss of cell or lineage identity and gain of properties of embryonic neural cells. Cancer initiation may be the result of activation/upregulation/gain of function of just a single or a few neural genes/proteins, e.g. KRAS, CTNNB1, MYC, EZH2, etc. At this stage, cancer cells are more similar to their healthy counterpart cells, either in morphology or in gene expression profile. With the progression of cancer development, they further trigger a series of signaling cascades being required for subsequent cell survival, migration, metastasis, etc. The gradual activation of neural-specific signaling cascades is accompanied with gradual suppression of cell type- or tissue-specific genes, so as to allow neural gene expression. These changes eventually lead to the loss of identity of the original cell type, gain of the characteristics of embryonic neural cells, and assume the morphology that is very different from cells at the initial stage of cancer development. In different cancer types, different internal/external interactions or crosstalk should be required for the activation/upregulation/gain of function of probably a distinct subset of neural genes that facilitate cancer progression in a cell- or tissue-specific environment. Since the intrinsic/extrinsic signals for tumorigenesis are different from those for normal neural specification/development, tumorigenesis should be an awry process of neural specification/development. If we scrutinize again cancer ‘EMT’, the expression change of ‘EMT’ markers and each event regulating ‘EMT’ actually portray small areas of the broad landscape for non-neural to neural transition during tumorigenesis, but not epithelial to mesenchymal transition. The malignant traits that have been ascribed to cancer ‘EMT’ are actually the properties of neural precursor/progenitor cells. Tumors also occur in the nervous system. This type of tumor formation does not require the suppression of non-neural or epithelial genes, and might be just a consequence of a broken balance between signals promoting neural development and signals restricting this process, via amplification or mutations of genes that play specific roles or are expressed specifically during neural development, most frequently, *MYCN*, *ALK*, *PHOX2B*, *AKT*, etc. [87, 88]. Figure 1 shows the proposed models for tumorigenesis.

Numerous traces for the relationship between neural specification/development and tumorigenesis or for the neural characteristics of cancer cells can be found from literatures. Besides extensive involvement of individual neural specification/development signals in the regulation of cancer initiation and progression (Refer [6] for a partial list of these literatures), nerve dependence in cancer has been noticed since 1946 [89]. Denervation generates a suppressive effect on several cancer types [90–92]. However, this dependence was just thought as a result of crosstalk between cancer and nerve [89, 93]. For example, cancer cells exhibit intrinsic ability of active migration along axons [94], and can make use of innervating neural circuitry to promote cancer progression [95]. However, it is known that various neurotrophic growth factors/receptors are upregulated in cancer to promote cancer cell survival, proliferation, and invasion [96–102]. Besides autocrine expression of neurotrophins/Trk receptors in cancer cells, many cancer types, such as pancreas, stomach, colon or prostate, show increased density of nerve [92, 103–105]. Autonomic nerve development was observed during prostate cancer progression [90, 106]. Colorectal cancer can produce neuronal cells, and neurogenesis marks aggressive tumor behavior and poor patient outcomes [104]. Moreover, gastric and colorectal cancer stem cells exhibit the potential of producing neuronal cells, which support cancer progression [107]. Enrichment for stem and neural/neuronal genes in benign prostatic samples is an indicator of higher aggressiveness [104, 108], and melanoma development seems to be a re-emergence of the state of neural crest progenitors [109]. Furthermore, cancer therapy can generate a side effect of neurodegeneration in both children and adults by disrupting normal neural stem and precursor cell function, leading to ultimately neurocognitive deficits in patients with tumors, including breast cancer, colorectal cancer, lymphoma, and brain tumors [110–113]. For example, methotrexate (MTX) is used for chemotherapy via targetting DHFR. Dhfr transcript is enriched in embryonic neural tissues [114, 115]. Moreover, MTX also influences BDNF [113], a factor that is expressed specifically in embryonic neural progenitor cells, regulates extensively adult neurogenesis and neuroplasticity, and plays a promoting role in cancer. These reports suggest that neural progenitor/stem cells have similar response to cancer therapy as cancer cells, providing a further association between cancer cells and neural cells. If these traces are integrated together, the evidence for intrinsic link between tumorigenesis and neural specification/development is quite obvious.

**Fig. 2:**
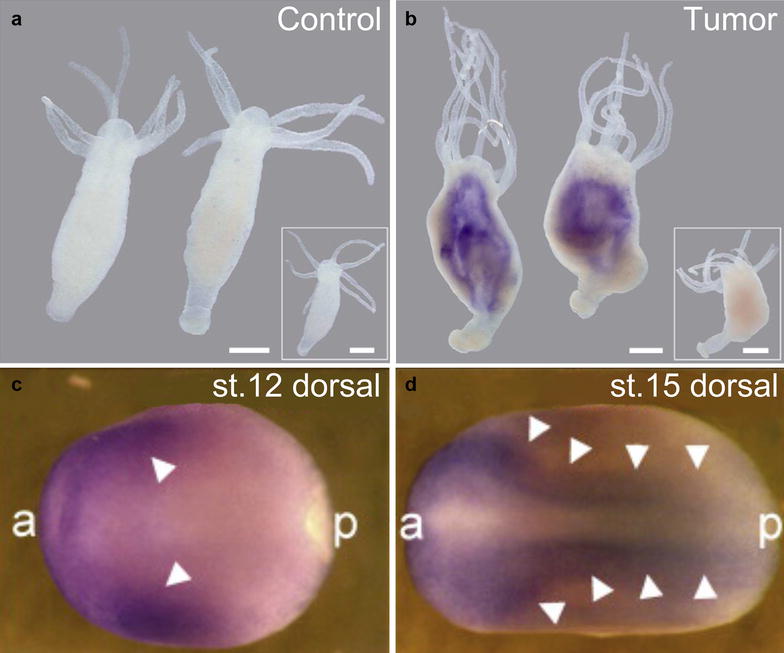
The link between gene conservation, tumorigenesis and neural development. **a**, **b** *tpt1* is not transcribed in control animals of *Hydra* (**a**); whereas in animals bearing tumor, it is strongly expressed and expression is localized to tumor polyps (**b**) as detected with whole mount in situ hybridization using a *tpt1* antisense riboprobe. Hybridization with a sense probe reveals no signal (insets). Scale bar, 300 μM. (**c**, **d**) *tpt1* transcription is localized to the neuroectoderm at the stage of neural induction (**c**) and to the neural plate and neural crest during neurulation (**d**) in *Xenopus laevis*, as shown by whole mount in situ hybridization. st.12 and st.15 refer to the Nieuwkoop and Faber stages 12 and 15 for *Xenopus* development. Arrowheads indicate domains of *tpt1* expression. *a* anterior, *p* posterior (**a**, **b** are adapted from [119] and **c**, **d** are from [120])

The neural specification/development model indicates that the process of tumorigenesis turns the cells of different lineages into a specific cell type. This is corroborated by that carcinogenesis represents a process of reverse evolution from multicellularity to unicellularity [116, 117]. One study also demonstrates that cancer related genes are mostly conserved in unicellular and basal species of multicellular organisms [3]. These studies together imply a connection between tumorigenesis, evolution and neural development. For an instance, TPT1 (translationally-controlled tumor protein. Also named TCTP, p23, fortilin, HRP) is overexpressed in human cancer and conserved from yeast to human [118]. Transcript coding for the homologous protein in the basal metazoan *Hydra* is localized to tumor polyps (Fig. 2b) [119]. Correspondingly, the homologous gene in the amphibian *Xenopus* is specifically expressed in neural plate and neural crest during neurulation in embryos (Fig. 2c, d) [120]. Evolution from unicellular to multicellular, and subsequently to higher organisms, is a process of diversification of cell or tissue types that execute different functions. Differentiation of different cell or tissue types is determined by the emergence of cell-type or tissue-specific genes. Therefore, loss of multicellularity is analogous to the loss of cell lineages and to dedifferentiation. Moreover, among the three germ layers, the ectoderm emerged the earliest during evolution, followed sequentially by endoderm and mesoderm [121]. This means that ectoderm cells are evolutionarily the closest to unicellular organisms. Coincidentally, the default state of ectodermal cells is neural [71, 72]. BMP signaling, a branch of TGFβ signaling pathway, is responsible for inhibition of neural fate and for specification of epidermal fate of ectodermal cells. TGFβ signaling and other signaling pathways that regulate basal differentiation events are present only in multicellular organisms [122, 123]. Hence, neural cells might be the cell type that is the closest to primitive unicellular organisms during evolution.

The extreme complexity of cancer has aroused many theories and hypothesis about cancer initiation and progression, typically the theories of somatic gene mutations, chromosomal instability, aneuploidy, speciation, mitochondrial dysfunction, Warburg effect, etc., each of them being partially logical and verifiable on the one hand and meeting with serious challenges on the other [4, 5]. A general theory accounting for an exclusive cause of cancer initiation and progression has long been sought [124]. Although new mutations, molecules or regulatory mechanisms, etc., are still being identified one after another, they seem to bring the field not closer to the goal of a unified theory, but serve primarily to upregulate the complexity. The advent of ‘omics’ studies boosts the complexity by piling up the data in a faster pace that leaves our capability far behind to interpret the meaning of these datasets in cancer biology [125]. The studies, from the finest details of individual nucleotides or amino acids to macroevolution [126, 127], or from classical oncogenes to microbes [128, 129] the very new player of cancer biology, etc., are reasonable in each individual cases, and each seems to be equally important for understanding cancer. However, most studies are focused on specific events of tumorigenesis and may not directly reflect a principle that exists in a different layer, which may be obscured by genetics [124] and revealed only by logical integration of information. Our work led us to the proposal that development of different cancer types represents the convergence of different cell lineages to a state that is characteristic of neural cells. This could be a framework for cancer initiation and progression. Gene mutations, chromosomal alterations, or changes in proteins or even microenvironment can each explain the initiation or progression of some cancer but not other, suggesting that these changes do not inevitably cause cancer. Moreover, these changes can be either the cause or the consequence of tumorigenesis, and can also be the cause or consequence of each other. No matter what situation it is, when signals for a neural specification process in a somatic cell are haphazardly triggered, cancer development might begin. It is logical that more frequent changes in genes, chromosomes, proteins, microenvironments, etc., can create a higher probability to trigger or promote neural specification process in non-neural cells.

## Conclusions

Cancer is featured by high heterogeneity. Numerous studies have revealed the minutest differences between cancer types or subtypes, or even between single cells [130–132]. However, these extreme specificities are perhaps related, if they are, to a very narrow window of the process of tumorigenesis. On the other hand, numerous genes, proteins or pathways seem to play equally important roles in regulating cancer initiation or progression. This raises the question what is the exact target we should aim at. Our study, in combination with numerous other studies, suggests that cancer initiation and progression may represent a process of gradual loss of original cell identity and gain of neural properties, providing a framework that unifies the malignant features of cancer cells. Within this framework, all the signals related to tumorigenesis can be grouped into two categories: the core signals and the peripheral signals. The core signals form regulatory networks to confer the characteristics of neural cells to cancer cells, whereas the peripheral signals may help to initiate, maintain or enhance the core signals in the internal/external environments specific to a cell or tissue type that compose cancer heterogeneity. Like that chopping off one or a few branches will not cause a critical damage to a tree’s life, targeting one or a few peripheral signals in cancer cells may also not be efficient for curbing the process of cancer progression. Even if a mutation may play a major role, for example KRAS (G12D) during cancer initiation, targeting the mutation itself will be probably not effective any more once the downstream signaling pathways have been stimulated and active during cancer progression. Moreover, chemoresistance is almost insurmountable in mechanism-based therapy due to several reasons, primarily the complicated signal feedback loops in cells [133]. ATP-dependent transporters also cause multidrug resistance in cancer cells [134]. The efficient approach to overcome these difficulties should be pinpointing the neural feature, i.e. targeting the regulatory networks as a whole instead of one or two signals, of cancer cells. In other words, cancer could be targeted as a cell type. Studies on several cancer types have shown that denervation suppresses cancer [90–92], providing some hints that support this strategy. The regulatory networks in cancer cells could be overturned as a whole by means of direct reprogramming/transdifferentiation using appropriate non-neural lineage specification factors or their combinations. This is not meant to turn cancer cells into another type of cells, but emphasizes the inhibitory effect on the overall neural regulatory networks in cancer cells by non-neural factors, thereby alleviating or eliminating significant signal feedback loops. One example could be that muscle cell specification factor MYOD can convert cancer cell lines into muscle-like cells [135] and inhibit RAS-induced cell transformation [79]. In summary, the information above might provide a stimulus for revisiting the molecular event-centered cancer research and considering focuses on the relationship between cell fate change and tumorigenesis.
